# Comparison of the benefits of cochlear implantation versus contra-lateral routing of signal hearing aids in adult patients with single-sided deafness: study protocol for a prospective within-subject longitudinal trial

**DOI:** 10.1186/1472-6815-14-7

**Published:** 2014-08-11

**Authors:** Pádraig T Kitterick, Gerard M O’Donoghue, Mark Edmondson-Jones, Andrew Marshall, Ellen Jeffs, Louise Craddock, Alison Riley, Kevin Green, Martin O’Driscoll, Dan Jiang, Terry Nunn, Shakeel Saeed, Wanda Aleksy, Bernhard U Seeber

**Affiliations:** 1 National Institute for Health Research (NIHR) Nottingham Hearing Biomedical Research Unit, Ropewalk House, 113 The Ropewalk, NG1 5DU Nottingham, UK; 2 Otology and Hearing group, Division of Clinical Neuroscience, School of Medicine, University of Nottingham, NG7 2UH Nottingham, UK; 3 Nottingham University Hospitals NHS Trust, Queen’s Medical Centre, NG7 2UH Nottingham, UK; 4 Midlands Hearing Implant Programme, Queen Elizabeth Hospital Audiology Centre, University Hospitals Birmingham, B15 2TH Birmingham, UK; 5 Central Manchester University Hospitals NHS Foundation Trust, Manchester Academic Health Science Centre, M13 9WL Manchester, UK; 6 University of Manchester, Oxford Rd, M13 9PL Manchester, UK; 7 Department of Audiology, St Thomas’ Hospital, Lambeth Palace Road, SE1 7EH London, UK; 8 The Royal National Throat, Nose and Ear Hospital, 330 Gray’s Inn Road, WC1X 8DA London, UK; 9 MRC Institute of Hearing Research, University Park, NG7 2RD Nottingham, UK; 10 Technische Universität München, Associated Institute Audio Information Processing, Arcisstrasse 21, 80333 Munich, Germany

**Keywords:** Cochlear implantation, Single-sided deafness, Unilateral hearing loss, Contra-lateral routing of signals, Hearing aid, Binaural hearing, Spatial listening

## Abstract

**Background:**

Individuals with a unilateral severe-to-profound hearing loss, or single-sided deafness, report difficulty with listening in many everyday situations despite having access to well-preserved acoustic hearing in one ear. The standard of care for single-sided deafness available on the UK National Health Service is a contra-lateral routing of signals hearing aid which transfers sounds from the impaired ear to the non-impaired ear. This hearing aid has been found to improve speech understanding in noise when the signal-to-noise ratio is more favourable at the impaired ear than the non-impaired ear. However, the indiscriminate routing of signals to a single ear can have detrimental effects when interfering sounds are located on the side of the impaired ear. Recent published evidence has suggested that cochlear implantation in individuals with a single-sided deafness can restore access to the binaural cues which underpin the ability to localise sounds and segregate speech from other interfering sounds.

**Methods/Design:**

The current trial was designed to assess the efficacy of cochlear implantation compared to a contra-lateral routing of signals hearing aid in restoring binaural hearing in adults with acquired single-sided deafness. Patients are assessed at baseline and after receiving a contra-lateral routing of signals hearing aid. A cochlear implant is then provided to those patients who do not receive sufficient benefit from the hearing aid. This within-subject longitudinal design reflects the expected care pathway should cochlear implantation be provided for single-sided deafness on the UK National Health Service. The primary endpoints are measures of binaural hearing at baseline, after provision of a contra-lateral routing of signals hearing aid, and after cochlear implantation. Binaural hearing is assessed in terms of the accuracy with which sounds are localised and speech is perceived in background noise. The trial is also designed to measure the impact of the interventions on hearing- and health-related quality of life.

**Discussion:**

This multi-centre trial was designed to provide evidence for the efficacy of cochlear implantation compared to the contra-lateral routing of signals. A purpose-built sound presentation system and established measurement techniques will provide reliable and precise measures of binaural hearing.

**Trial registration:**

Current Controlled Trials http://www.controlled-trials.com/ISRCTN33301739 (05/JUL/2013)

## Background

Permanent acquired unilateral severe-to-profound hearing loss, or single-sided deafness (SSD), has been estimated to affect between 12–27 individuals in every 100,000 of the general population with the majority of losses being sudden and idiopathic; that is, a cause has not or cannot be determined [1]. Other aetiologies which may give rise to SSD include Vestibular Schwannoma (and associated surgery) and Ménière’s disease. Despite having normal or near-normal hearing in one ear, individuals with SSD report difficulty when listening in many everyday situations [2,3]. In particular, patients report disability when localising sounds and listening to sounds on the side of the impaired ear [4]. Compatible with these self-reported difficulties, individuals with SSD show little or no ability to localise sounds [5-8] and display a poor ability to understand speech in noise when the speech is on the impaired side of the head [5,6]. Both impairments reflect a lack of access to binaural cues such as inter-aural time and level differences, while the latter impairment also reflects the acoustic shadow cast by the head which attenuates the high-frequency components of sounds at the ear contra-lateral to their source.

In the UK National Health Service (NHS), the standard of care for SSD is a Contra-lateral Routing of Signals (CROS) hearing aid system. A CROS system picks up sounds arriving at the impaired ear using a remote microphone and presents those sounds to the non-impaired ear through a wired or wireless link [9]. The primary function of the system is therefore to overcome the acoustic shadow cast by the head and, in doing so, to improve access to sounds on the impaired side. The use of a CROS system has been found to improve the perception of speech in noise compared to the unaided condition when the most favourable signal-to-noise ratio is available at the impaired ear; i.e. with speech on the impaired side and the noise from the front [6,10,11] or speech from the front and noise on the non-impaired side [7,12,13]. Use of a CROS system has also been associated with a reduction in self-reported difficulty with background noise, communication, and reverberation [1]. However, the indiscriminate routing of sounds from the impaired ear to the non-impaired ear can produce undesirable results. For example, the perception of speech in noise can degrade with CROS use compared to the unaided condition when a background noise is located on the impaired side [6,11,13]. The use of a CROS has also been found to have no effect on localisation accuracy [6,10,11] which is compatible with the fact that the device is not designed to restore access to the binaural cues which underpin the ability to locate sounds in space. Finally, patients also report dissatisfaction with the requirement to wear a hearing aid device on their non-impaired ear [12,14].

Alternatives to a CROS include a Bone-Anchored Hearing Device (BAHD) which also transmits sounds arriving on the impaired side to the non-impaired ear but achieves this by conduction through the cranial bones. There is some emerging evidence that BAHD may provide benefits to speech perception in noise over a CROS system although more high quality trials are needed [1,15]. BAHD is currently only commissioned in the NHS in cases of profound unilateral sensorineural hearing loss where conventional acoustic aiding (such as with a CROS) is not possible or contra-indicated [16]. Like the CROS, a BAHD does not restore binaural hearing in individuals with SSD for whom the sensorineural component of the hearing loss is severe-to-profound. Poor localisation ability has been cited by patients as a factor which contributes to their decision not to receive a BAHD, as have cosmetic concerns about the placement of a permanent bone-anchored abutment through the skin [17].

An alternative treatment for SSD which does have the potential to restore access to binaural cues is cochlear implantation. In cases of SSD where the auditory nerve is intact, impairments of the middle and inner ear can be bypassed and the auditory nerve stimulated electrically. Like CROS and BAHD devices, cochlear implantation in SSD has been found to improve access to sounds on the impaired side and has been associated with a reduction in self-reported difficulties with listening to speech in noise [5,18,19]. Unlike CROS and BAHD devices, cochlear implantation has also been found to improve the accuracy with which sounds can be localised [5,18,19]. Thus, the restoration of input to the impaired ear has the capacity to provide access to the inter-aural cues which support localisation and to restore useful aspects of binaural hearing. The intervention therefore has the potential to alleviate the high-levels of difficulty that individuals with SSD report with understanding speech in many everyday situations containing background noise [4] and, in doing so, may improve their health-related quality of life [20].

In the NHS, cochlear implantation is currently commissioned for adults and children with bilateral severe-to-profound deafness [21] and is not commissioned routinely for individuals with SSD. Providing a cochlear implant in cases of SSD and supporting its effective use throughout the lifespan is likely to incur a substantially greater cost to the NHS compared to the provision and maintenance of the current standard of care, a CROS system. A cochlear implant is also a lifetime commitment and involves a surgical procedure whereas a CROS system is no more permanent or invasive than a conventional acoustic hearing aid. For these two reasons, it was anticipated that cochlear implantation would be unlikely to be considered as an *alternative* to CROS in the NHS. Rather, it may be considered as an intervention for SSD in cases where a CROS system has been found to offer insufficient benefit as in these cases no other treatment options are currently available on the NHS. The current trial was therefore designed to evaluate whether cochlear implantation provides benefit to those patients who report receiving insufficient benefit from a CROS system.

### Purpose

This within-subjects longitudinal trial will determine whether cochlear implantation provides significant benefit in patients who have failed to receive sufficient benefit from a Contra-lateral Routing of Signals (CROS) system.

### Primary objectives

• Does a cochlear implant significantly improve sound localisation compared to a CROS hearing aid in patients who report insufficient benefit from a CROS?

• Does a cochlear implant significantly improve speech perception in noise compared to a CROS hearing aid in patients who report insufficient benefit from a CROS?

### Secondary objective

• Does a cochlear implant significantly improve quality of life compared to a CROS hearing aid in patients who report insufficient benefit from a CROS?

## Methods/Design

**Figure 1:**
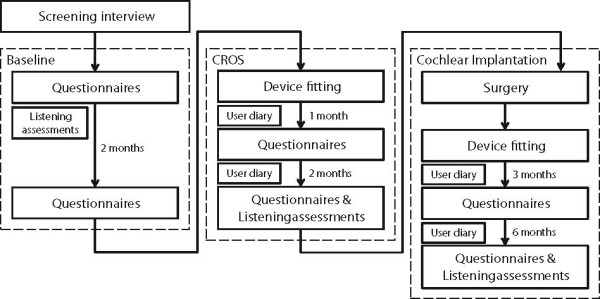
**Trial design.** A schematic representation of the sequence and timing of appointments that each participant will complete divided into the three trial phases. In the baseline phase, participants will use no hearing-assistive device and the listening tests will be administered during the two-month delay between baseline questionnaire assessments. After both CROS and cochlear implant fitting, participants will complete a daily diary to track device usage and to capture any comments on the advantages/disadvantages of the devices. CROS: Contra-lateral Routing of Signals.

The trial is a within-subjects longitudinal study in which participants will be assessed before and after receiving two interventions delivered in a fixed order. Participants will be recruited from five NHS Trusts in the UK: (1) Nottingham University Hospitals NHS Trust; (2) University Hospitals Birmingham NHS Foundation Trust; (3) Central Manchester University Hospitals NHS Foundation Trust; (4) University College London Hospitals NHS Foundation Trust; and (5) Guy’s and St. Thomas’ NHS Foundation Trust. The trial is being coordinated by the National Institute for Health Research (NIHR) Nottingham Hearing Biomedical Research Unit, Nottingham. After screening against the inclusion/exclusion criteria, participants will progress through three phases: baseline, CROS, and cochlear implantation (Figure 1). During the two-month baseline phase, participants receive no treatment; in the CROS phase, participants will be fitted with a CROS system and complete a 3-month trial of the device; in the cochlear implantation phase participants will receive a cochlear implant and follow-up will continue for 9 months.

The duration of the CROS trial (3 months) was chosen in consultation with clinical colleagues from several UK audiology services to ensure that (a) it was of sufficient duration to allow the benefits from CROS to emerge; (b) any benefits from CROS would have stabilised; and (c) self-reported benefits would be based on patients’ experience of CROS use rather than any pre-conceived expectations (whether positive or negative). The duration of the cochlear implantation follow-up period (9 months) was chosen based on data from previous studies which have observed that (a) a large proportion of the benefit from cochlear implantation is achieved within the first 9 months and additional benefits emerge at a slower rate over the course of several years [22,23]; and (b) significant benefits to binaural hearing from cochlear implantation in patients with SSD can be achieved as early as 6 months after implantation [5].

The primary endpoints are the assessments of sound localisation and speech understanding in noise at baseline, after CROS aiding, and after cochlear implantation. Secondary endpoints are questionnaire measures of hearing- and health-related quality of life and the impact of tinnitus at baseline, after CROS aiding, and after cochlear implantation.

The trial protocol and the study activities across the five NHS trusts were given a favourable opinion by the National Research Ethics Committee, East Midlands Nottingham - 2, Nottingham, UK (12/EM/0378). The sponsor of the trial is Nottingham University Hospitals NHS Trust, Research and Innovation Department, Nottingham, UK.

### Population and sample size

Participants will be recruited from a notes review at each of the participating hospitals. The primary outcome measure is sound localisation accuracy; specifically, the mean absolute angular error in localising speech sounds. The trial is powered to detect a within-subject improvement in mean localisation accuracy resulting from the use of a cochlear implant compared to, and following the use of, a Contra-lateral Routing of Signals (CROS) hearing aid. A previous study examined the within-subject improvement in localisation accuracy after cochlear implantation compared to brief periods of CROS and simulated BAHD use prior to implantation [5]. The authors reported localisation performance after 3-week trials of both a CROS aid and a BAHD on a soft band (without a permanent bone-anchored abutment), completed in a random order. They subsequently reported localisation performance data obtained 6 months after participants had received a cochlear implant.

Arndt et al. [5] reported the median, range, and inter-quartile range of localisation performance at the end of the follow-up period for each device. Standard deviations for the localisation performance data after 3 weeks of CROS use and after 6 months of cochlear implant use were estimated from the inter-quartile ranges [24]. As only summary statistics were available, the correlation between the CROS (i.e. pre-cochlear implant) and post-cochlear implant measures could not be determined. The data were assumed to be uncorrelated yielding an estimated effect size of 1.63 standard deviations. To detect an effect of this magnitude using a one-tailed Wilcoxon signed-rank test at *α*=0.05 and with 95% power would require a sample size of 7. A final sample size of 10 was chosen to achieve the desired statistical power and to allow for some attrition due to the length of the follow-up period after cochlear implantation (9 months). The desired power reflects the fact that a study of this nature is unlikely to be repeated due to the substantial cost of providing the participants with a cochlear implant.

#### *Inclusion criteria*

• Eighteen years of age or older at the time of entry to the trial

• Able and willing to undertake all assessments required by the trial

• Good understanding of written and spoken English

• Poorer ear (ear to be implanted): 

– Acquired (post-lingual) severe-to-profound sensorineural hearing loss of <10 years duration

– Severe-to-profound deafness defined as having hearing threshold >90 dB HL at 1 & 4 kHz, and >65 dB at 0.5 kHz

– Minimal benefit from a hearing aid (see below)

• Better ear (contra-lateral ear): Normal or near-normal hearing. For the purposes of this trial this is defined as hearing thresholds with a pure-tone average (PTA) of ≤30 dB HL at 0.5, 1, & 2 kHz.

**Figure 2:**
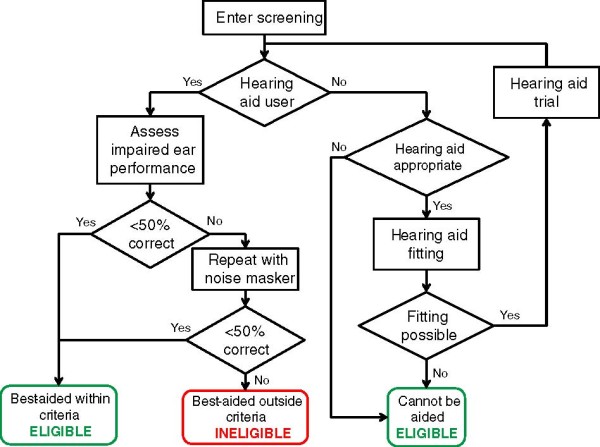
**Functional screening assessment.** A flow chart of the screening process concerned with assessing potential participants against the inclusion criterion governing the acceptable level of function in the impaired ear. If aidable, functional performance will be assessed while the NH ear is occluded using an earplug and circumaural mufflers. Should the level of function still fall outside criterion, performance will be assessed again but while a masking noise is also presented to the NH ear. Individuals who cannot be aided, either because it is deemed clinically inappropriate or because it is not possible fit an aid (e.g. few or no measurable audiometric thresholds), will be eligible for inclusion. NH: Normal Hearing.

The process that will be used to determine minimal benefit from a hearing aid is illustrated in Figure 2. For those patients who use a hearing aid at the time of the screening interview, speech perception performance in the best-aided condition will be assessed using a method similar to that used for traditional cochlear implantation candidates [21]. Two lists of Bamford-Kowal-Bench (BKB) sentences will be presented at 70 dB Sound Pressure Level from a loudspeaker positioned in front of the patient while their normal hearing ear is occluded using a combination of an earplug and a circumaural muffler. Performance will be measured in terms of the proportion of keywords reported correctly. If less than 50% of keywords are reported correctly, the patient will be considered as receiving minimal benefit from their hearing aid. If the percentage of keywords reported correctly is 50% or greater, performance will be reassessed using two different BKB lists while the function of the normal hearing ear is further degraded using a combination of a masking noise presented at 50 dB(A) via an insert tube-phone and a circumaural muffler. If less than 50% of the keywords are reported correctly with masking, the patient will be considered as receiving minimal benefit from their hearing aid.

For those patients who do not use a hearing aid at the time of the screening interview, an assessment will be made as to whether it would be clinically appropriate to provide amplification in the impaired ear. This assessment process will reflect current standard clinical practice. If the provision of a hearing aid would be clinically appropriate, the patient will be referred for hearing aid fitting to confirm whether the level of gain being called for can be obtained using any of the hearing aid models available on the NHS, and whether the prescription can be achieved. Verification of the prescription will be determined using real-ear measurements [25]. If the necessary gain can be provided and the prescription can be achieved, the patient will undergo a trial of a hearing aid at their local audiology centre before speech perception is assessed in their best-aided condition as described above. The duration of the trial will vary according to local practice but will last for a minimum of four weeks. If amplification is deemed inappropriate, or the necessary gain cannot be provided or achieved, the patient will be deemed to be unaidable and will be considered for inclusion in the trial.

Participants may have previous experience with using a CROS system. However, participants must have ceased use of any CROS system prior to entry into the study so that they may be evaluated in their unaided state during the baseline phase.

#### *Exclusion criteria*

• Evidence of middle-ear pathology based on otologic examination and tympanometry

• Medical or psychological conditions that contraindicate undergoing surgery

• Tinnitus as primary motivation for treatment

• Ossification or any other cochlear anomaly that might prevent complete insertion of the electrode array

• Hearing loss of neural or central origin, including auditory neuropathy and neurofibromatosis type II

• Additional handicaps that would prevent or restrict participation in the audiological evaluations

• Unrealistic expectations on the part of the participant regarding the possible benefits, risks, and limitations which are inherent to the surgical procedure and prosthetic device

• Unwillingness of the participant to comply with all of the trial requirements

• Any known factor which would limit the benefit obtainable from a cochlear implant

A participant will be withdrawn from the trial if there is a failure of the cochlear implant device or they experience a serious or intolerable device-related adverse event leading to the explant or discontinued use of the cochlear implant device. Participants may withdraw themselves from the trial at any time and without giving a reason. Participants who withdraw voluntarily during the CROS or cochlear implantation phases may continue to use their current device (CROS or cochlear implant) indefinitely.

### Interventions

#### *Contra-lateral routing of signals*

The CROS hearing aid comprises a conventional acoustic hearing aid and a remote microphone. The remote microphone is worn on the impaired ear and the hearing aid is worn on the non-impaired ear. Sounds arriving at the impaired ear are picked up by the remote microphone and sent via a wireless link to the hearing aid which delivers the sounds via air conduction to the non-impaired ear. The acoustic coupling for the hearing aid is selected to have the smallest possible impact on the sound arriving at the non-impaired ear.

The trial will use the Phonak CROS system (Phonak AG, Stäfa, Switzerland). The system will be fitted by an experienced audiologist using the standard fitting software provided by Phonak (Target™3.0). The fitting procedure ensures that the acoustic information at the non-impaired ear is similar regardless of whether a sound source is incident to the impaired or non-impaired ear [26,27]. The fit will be verified using real-ear measurements. At the time of fitting, participants will be encouraged to use the CROS system for as long as possible each day and to record the number of hours of use in their user diary each day. Participants will attend a follow-up appointment after one month of CROS use during which their initial usage will be assessed, additional encouragement will be given, and the fitting adjusted as required.

#### *Cochlear implant*

A cochlear implant comprises an externally-worn sound processor and a wholly-implantable receiver-stimulator. The receiver-stimulator is connected to a micro-electrode array that is surgically-inserted into the inner ear (cochlea). Sounds are picked up by one or more microphones in the sound processor and are separated into frequency bands containing both slow changes in amplitude over time (envelope) and rapid changes in frequency around the centre frequency of each band (fine structure). The sound processor extracts the envelope in each frequency band and uses the envelopes to define the pattern of pulses to be delivered by each electrode. The pulse patterns are delivered through the scalp to the implanted receiver-stimulator via a radio-frequency coil.

The trial will use the Cochlear^TM^ NucleusⓇ Cochlear Implant System (Cochlear Ltd, NSW, Australia). The NucleusⓇ CI442 implant will be implanted by experienced ENT surgeons. The implant system will be activated once the wound has healed, typically 3–6 weeks after surgery, and the sound processor will be fitted by an experienced cochlear implant audiologist. The programme of post-activation fitting and rehabilitation appointments will reflect the current standard care delivered to traditional cochlear implant candidates in the UK. Participants will be encouraged to use their cochlear implant for as long as possible each day and to record their actual usage in their user diary. Participants will also be encouraged to follow a rehabilitation programme which will include listening to speech materials fed directly into the sound processor of the implant via an accessory cable.

### Device fitting procedures

#### *CROS*

The CROS fitting procedure aims to preserve the resonance characteristics of the non-impaired ear and to overcome the head shadow which would otherwise attenuate sounds arriving at the impaired ear. The procedure requires the use of Real Ear Measurements (REMs). REMs will be carried out according to recommendations from the British Society of Audiology [25] and manufacturer-specific instructions based on the fitting hardware and software being used. This will include the choice of stimulus type, equipment setup, fitting environment, participant positioning, and probe calibration method.

The probe tube will be calibrated prior to commencing the fitting procedure. The procedure involves making three probe measurements [26]: 

1. A real-ear unaided response (REUR) while the loudspeaker is positioned 45 degrees towards the non-impaired ear. The probe tube and reference microphone are both situated at the non-impaired ear. The CROS system is not worn during this measurement.

2. A real-ear aided response (REAR 1) measured using the same setup as for the REUR. The CROS system is worn and turned on during this measurement.

3. A second real-ear aided response (REAR 2) measured while the loudspeaker is positioned 45 degrees towards the impaired ear. The probe tube is still situated at the non-impaired ear but the reference microphone is situated at the impaired ear.

The measurement REAR 1 will be compared to the REUR to confirm that the coupling of the hearing aid to the non-impaired ear is largely acoustically transparent. Any gross dissimilarity will be addressed by checking and reconsidering the method of acoustic coupling. The REAR 1 and REAR 2 measurements will be compared and checked to be within ±3 dB across the available frequency range. Any deviations between the two measurements will be addressed by adjusting the gain of the CROS system and repeating the measurement of REAR 2.

The available fitting hardware and software at each of the five trial sites may not permit the separation of probe tube and reference microphone on opposite sides of the head as required for the measurement of REAR 2. An alternative procedure has been described based on a stored equalisation method [27] and will be used where probe and reference cannot be separated.

#### *Cochlear implant*

The cochlear implant fitting procedure will follow the manufacturer-recommended procedure for fitting using the Custom SoundⓇ software. The default programming parameters specified by the manufacturer will be used unless the individual needs of a patient require a deviation from those parameters to maximise benefit. In general terms, the fitting procedure will involve checking electrode impedances, disabling electrodes based on impedance measurements and/or intra-operative findings, creating a mapping between input frequency bands and electrodes (MAP), and establishing the minimum detectable (threshold) stimulation level and the maximum comfortable stimulation level for each electrode.

Cochlear implant fitting is an iterative process to maximise hearing function, reflecting the emergence of hearing function over time due to a gradual acclimatisation and adaptation to electrically-delivered information. Participants will therefore attend several programming sessions in the initial months after activation of their implant through which their MAP, threshold levels, and comfort levels will be fine tuned to maximise speech understanding and acceptability to the patient.

### Primary outcome measures

Assessments of the ability to localise sounds and understand speech in noise will be administered using a purpose-built test setup [28-30] in an anechoic chamber at the MRC Institute of Hearing Research in Nottingham.

#### *Sound localisation*

**Figure 3:**
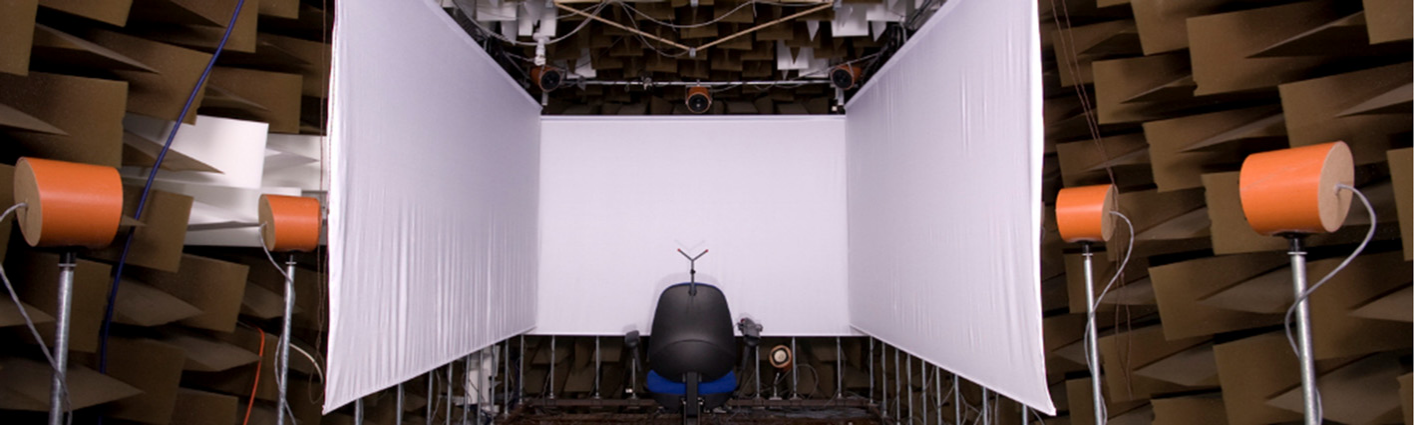
**Testing apparatus.** The apparatus that will be used to assess the sound localisation accuracy and speech understanding of patients in the trial. An array of loudspeakers and a visual projection system are positioned within an anechoic chamber. Thirty-six loudspeakers are distributed horizontally around the listening position with an additional 6 loudspeakers suspended above the listener and a further 6 loudspeakers positioned below the listener. The three visual projection screens are driven by three high-definition projectors. Responses are recorded using a trackball (localisation task) or through an intercom (speech understanding task).

Sound localisation will be measured in anechoic conditions and a range of reverberant conditions following established methods using a visual-pointing technique [31]. On each trial, a short pre-recorded speech segment will be presented from one of several loudspeakers positioned in the frontal plane and behind an acoustically-transparent curtain (Figure 3). Speech segments will be short isophonemic words [32] spoken by a male talker with a British accent. The task of participants will be to indicate the perceived location of the sound by moving a visual pointer projected onto the curtain. The position of the visual pointer will be adjusted using a computer-controlled trackball mouse and with a precision exceeding one degree of visual angle. Presentation levels will be roved from trial to trial to restrict the use of monaural loudness cues and limited so that stimuli do not engage the compression features of the cochlear implant sound processor which can degrade inter-aural level cues [33-35].

The original speech waveforms will be presented after being convolved with the room impulse of a simulated room, the methods and parameters of which have been described previously [30]. The ratio between the direct and reverberant sound will be varied to simulate different room characteristics from anechoic (no reflections) to highly-reverberant. Localisation accuracy in anechoic conditions will reflect the ability of participants to access and use monaural spectral cues and inter-aural cues under ideal listening conditions. Comparison of localisation in anechoic conditions and in reverberant conditions will identify the level of reverberation that participants can tolerate while maintaining accuracy. Summary measures of localisation accuracy will be reported for each individual participant along with measures of bias and variance in their responses. The trial is powered using localisation data from a previous before-after study of cochlear implantation following CROS and simulated BAHD use [5].

#### *Speech understanding in noise*

Speech understanding in noise will be measured in terms of a speech-reception threshold (SRT) which represents the signal-to-noise ratio (SNR) at which 50% of sentences are reported correctly. Sentences will be drawn from a recording of the IEEE sentence corpus [36] spoken by a female talker with a British accent. The masking noise will be generated to match the long-term average spectrum of the sentences. SRTs will be measured using an adaptive procedure to avoid floor and ceiling effects by varying the level of the speech and noise from trial to trial based on the accuracy with which the participant recalls keywords in the sentences [37]. The spatial configuration of the speech and the noise will be varied across testing conditions to estimate the three main effects of two-eared listening to speech in noise [38]: i) the head-shadow effect estimated from the test conditions in which the speech is positioned on the side of the impaired ear and the noise is on the side of the non-impaired ear; ii) binaural squelch effect estimated from the test conditions in which the noise is positioned on the side of the impaired ear and the speech is on the side of the non-impaired ear; iii) binaural summation (redundancy) effect estimated from the test conditions in which the speech and noise are positioned equidistant from both ears; i.e. directly in front of the participant. SRTs will be reported for each individual participant along with a measure of the variance of the SNRs used to compute each SRT.

### Secondary outcome measures

#### *Hearing-specific quality of life questionnaires*

The Speech, Spatial and Qualities of Hearing Scale (SSQ) [39] contains 47 questions organised into three sections. The *Speech* section asks about difficulties with understanding speech in a range of everyday situations. The *Spatial* section asks about difficulties with localising sound sources and tracking moving sounds. The *Qualities* section asks about difficulties with the perceived naturalness of sounds, separating sounds, and the amount of effort required to listen. The SSQ has been found to be capable of distinguishing between groups of unilateral and bilateral hearing aid [40] and cochlear implant [41] users, confirming its sensitivity to spatial hearing abilities, and found to have a test-retest reliability of 0.83 [42].

Glasgow Hearing Aid Benefit Profile (GHABP) has been developed as a patient-centred outcome measure designed to measure the disability caused by a patient’s hearing loss before and after hearing aid fitting and the impact that the disability has on a patient’s everyday life [43,44]. The GHABP asks six questions about four specific listening situations and up to four listening situations nominated by the patient. Each question is a five-point Likert scale. The six questions correspond to the six domains of initial disability, impact on life (handicap), device use, device benefit, residual disability, and device satisfaction.

The Glasgow Benefit Inventory (GBI) is an instrument for measuring changes in quality of life as a result of otorhinolaryngological interventions [45]. The GBI is designed to be administered after the intervention has been received and provides a direct measure of its effect on the health state of the patient. The 18 questions are grouped to form three sub-scales: general, social support, and physical health. The GBI has been found to be responsive to unilateral cochlear implantation [46].

Tinnitus Functional Index (TFI) has been designed to measure treatment-related changes in tinnitus intrusiveness and severity. The development of the TFI has included the definition of the minimum clinically-important change in TFI score (13 points) and identified a test-retest reliability of 0.78 [47].

A user diary will also be provided in booklet form for the 3-month trial of the CROS system and for the 9-month follow-up period after cochlear implantation. Participants will be instructed to record the duration of their device use on a daily basis and will also be encouraged to record any comments about situations in which either intervention was particularly helpful or unhelpful.

#### *Generic quality of life questionnaires*

The Health Utilities Index Mark 3 (HUI3) is a generic preference-based method for describing health status and measuring health-related quality of life [48]. The HUI3 health status classification system describes an individual’s health state on eight dimensions of health: vision, hearing, speech, ambulation, dexterity, emotion, cognition, and pain. The utility value for a chronic health state described using the classification system can be calculated using the HUI3 multi-attribute utility function. The multi-attribute function has been derived based on the preferences of a sample of the Canadian general public [49,50]. Utility values are defined by a scale on which death has a value of 0 and perfect or optimal health has a value of 1. Utility values derived using the HUI3 are suitable for use as part of a health-economic analysis. The HUI3 has been found to be responsive to interventions for hearing loss including acoustic hearing aids [51,52] and cochlear implants [53].

The EQ-5D is a generic method for describing and valuing health states [54,55]. The EQ-5D has two components. The first component is a system for describing a health state on five dimensions of health: mobility, self-care, usual activities, pain/discomfort, and anxiety/depression. A single utility value or ‘tariff’ for a health state can be calculated using a model which describes the preferences of a sample from the UK population [56]. The second component is a visual-analogue scale (VAS) used to obtain the patient’s own opinion of their current health state. The EQ-5D has been found to be unresponsive to changes in health-related quality of life arising from severe-to-profound deafness [57] and provides smaller estimates for the change in utility after cochlear implantation compared to the HUI3 [46]. However, the EQ-5D is the preferred instrument of the UK National Institute for Health and Care Excellence (NICE) for measuring health-related quality of life [58].

### Statistical methods, data analysis and reporting

Analysis of the trial data will include all patients who pass the inclusion and exclusion criteria and will be conducted on an intention-to-treat basis. Participants who are withdrawn at any stage will be replaced. Participants who choose to leave during the baseline or CROS phases of the trial will be replaced. Participants who choose to leave after receiving a cochlear implant will not be replaced. Data from participants who choose to leave will be retained and included in the analyses of individual trial phases; i.e. baseline, CROS, and cochlear implantation. Due to the small sample size, missing data will not be imputed when conducting within-subject comparisons of the incremental change from baseline to CROS and from CROS to cochlear implantation. Drop-out rates at each phase of the trial will be reported as a marker of potential bias in the between-phase comparisons.

Baseline characteristics of the participants will be reported including (but not limited to) age, gender, audiometric thresholds, aetiology and history of hearing loss, duration of severe-to-profound deafness, and history of hearing aid use.

### Efficacy analysis

The primary endpoint and analysis of the trial data will be at the end of the 9-month follow-up period after cochlear implantation. The analysis will compare outcomes in the cochlear implant phase to outcomes in the CROS phase. Outcomes in the CROS and baseline phases will also be compared. Comparisons will be performed for both the primary and secondary outcome measures. Comparisons both within and between trial phases will be performed using paired Student’s *t*-tests, Wilcoxon signed-rank tests, and repeated-measures analysis of variance, as appropriate. For each outcome measure, summary measures of the size and variance in the incremental benefit from cochlear implantation (difference between cochlear implantation and CROS) and CROS (difference between CROS and baseline) will be reported.

### Safety analysis

The reporting of adverse and serious adverse events will follow the standard operating procedure (SOP) for trials of medical devices specified by the sponsor organisation (Nottingham University Hospitals NHS Trust SOP 52).

## Discussion

Self-report and behavioural data confirm that individuals with SSD experience difficulties with listening to sounds on the side of their impaired ear and in determining the location of sounds in space [2-4]. Current treatment options for SSD primarily aim to improve access to sounds arriving at the impaired ear by utilising the intact hearing in the contra-lateral ear. Sounds at the impaired ear are transmitted to the non-impaired ear either via a wireless link and a conventional acoustic coupling (CROS) or by conduction via the cranial bones (BAHD). Individually, both systems have been found to be efficacious compared to an unaided condition in improving speech perception in noise but the systems do not improve the ability to localise sounds [1]. Cochlear implantation in SSD can restore the ability to localise sounds [5,18,19] by providing access to the inter-aural cues which underpin accurate localisation [35] and thus has the potential to support useful aspects of binaural hearing.

The current trial has been designed to provide evidence for the efficacy of cochlear implantation as an intervention in SSD compared to the current standard of care, a CROS system. A cochlear implant will be offered to those who receive insufficient benefit from a CROS system and for whom few other treatment options currently exist on the UK NHS. Participants will be evaluated prior to any intervention (baseline), after 1 and 3 months of CROS use, and at 3 and 9 months after cochlear implantation. The use of established measurement techniques [31] and a sound presentation system purpose-built for assessing speech perception in noise and sound localisation accuracy [28] will provide high-quality and reliable measures of binaural hearing; all patients will be tested in the same centre to reduce variability and improve trial consistency. The use of generic- and hearing-related instruments to assess health-related quality of life will provide estimates of the incremental change in quality of life after cochlear implantation to inform future trials of the cost-effectiveness of cochlear implantation in SSD.

## Trial status

The trial is currently in recruitment phase.

## Abbreviations

BAHD: Bone-Anchored Hearing Device; CROS: Contra-lateral Routing Of Signals; HUI3: Health Utilities Index Mark 3; GBI: Glasgow Benefits Inventory; GHABP: Glasgow Hearing Aid Benefit Profile; NHS: National Health Service (UK); NIHR: National Institute for Health Research; NRES: National Research Ethics Service; PTA: Pure-Tone Average; REUR: Real-Ear Unaided Response; REAR: Real-Ear Aided Response; REM: Real Ear Measurement; SD: Standard Deviation; SOP: Standard Operating Procedure; SNR: Signal-to-Noise Ratio; SRT: Speech-Reception Threshold; SSQ: Speech, Spatial and Qualities of Hearing Scale; TFI: Tinnitus Functional Index; VAS: Visual-Analogue Scale.

## Competing interests

This study is supported by infrastructure funding from the National Institute for Health Research (NIHR). PTK, GMOD and BUS were awarded an industry grant from Cochlear Europe Ltd (Surrey, UK) to cover the surgical, device, and rehabilitation costs of cochlear implantation for participants in the trial. The CROS hearing aids are being provided by Phonak Group Limited (Warrington, UK).

## Authors’ contributions

All authors (PTK, GMOD, MEJ, AM, EJ, LC, AR, KG, MOD, DJ, TN, SS, WA, BUS) developed the protocol. PTK, GMOD, MEJ and BUS drafted the manuscript. All authors read and approved the final version.

## Pre-publication history

The pre-publication history for this paper can be accessed here:

http://www.biomedcentral.com/1472-6815/14/7/prepub
